# Mechanically Induced Motor Tremors Disrupt the Perception of Time

**DOI:** 10.1523/ENEURO.0013-24.2024

**Published:** 2024-09-13

**Authors:** Keri Gladhill, Rose De Kock, Weiwei Zhou, Wilsaan Joiner, Martin Wiener

**Affiliations:** ^1^ George Mason University, Fairfax, Virginia 22030; ^2^University of California, Davis, Davis, California 95616

**Keywords:** cue combination, motor movements, time perception

## Abstract

Contemporary research has begun to show a strong relationship between movements and the perception of time. More specifically, concurrent movements serve to both bias and enhance time estimates. To explain these effects, we recently proposed a mechanism by which movements provide a secondary channel for estimating duration that is combined optimally with sensory estimates. However, a critical test of this framework is that by introducing “noise” into movements, sensory estimates of time should similarly become noisier. To accomplish this, we had human participants move a robotic arm while estimating intervals of time in either auditory or visual modalities (*n* = 24, ea.). Crucially, we introduced an artificial “tremor” in the arm while subjects were moving, that varied across three levels of amplitude (1–3 N) or frequency (4–12 Hz). The results of both experiments revealed that increasing the frequency of the tremor led to noisier estimates of duration. Further, the effect of noise varied with the base precision of the interval, such that a naturally less precise timing (i.e., visual) was more influenced by the tremor than a naturally more precise modality (i.e., auditory). To explain these findings, we fit the data with a recently developed drift-diffusion model of perceptual decision-making, in which the momentary, within-trial variance was allowed to vary across conditions. Here, we found that the model could recapitulate the observed findings, further supporting the theory that movements influence perception directly. Overall, our findings support the proposed framework, and demonstrate the utility of inducing motor noise via artificial tremors.

## Significance Statement

Our perception of time is naturally tied to movements of the body. Yet, how bodily movements bias or enhance estimates of time is still not well understood. We recently proposed that time estimates from body movements are combined with those from other sensory modalities via a Bayesian cue combination mechanism. This suggests that, by adding noise into body movements, time estimates from other sensory modalities should also be noisier. Here, we find evidence for this effect across two experiments where human subjects judged intervals of time while moving a robotic arm at different levels of tremor. These findings support the connection between body movements and time and provide an additional avenue of research using noisy movements to impact sensory estimates.

## Introduction

An abundance of previous research indicates that movement parameters differentially affect time estimates—in some cases movement biases the perception of time, whereas in other cases, it improves the precision of time estimates. It has recently been suggested that these contrasting effects can be explained via a Bayesian cue combination framework in which, along with sensory input such as auditory and visual information, movement itself also serves as an additional input for duration information (De Kock et al., 2021a). This work builds on previous research suggesting that time perception arises through our understanding of actions and their consequences (Merchant and Yarrow, 2016; Coull and Droit-Volet, 2018). Similarly, recent work has suggested that timing in animals may arise exclusively through movements, supporting a close connection between the motor system and time perception (Robbe, 2023).

Evidence for this framework comes from work over the past decade demonstrating the impact of movements on time estimates. In particular, we have shown that, when subjects are allowed to freely move a robotic arm, as opposed to having the arm restrained in place, their estimation of concurrently presented sensory time estimates is more precise (Wiener et al., 2019). This effect occurs both within and between subjects, across different task designs, and does not depend on the type of movement strategy employed. Rather, the effect appears to depend on whether the subject is moving or not. In a further series of experiments, we additionally found that increasing the viscosity of movements, such that movement lengths were shortened, also shortened time estimates (De Kock et al., 2021b). Furthermore, this effect was tied to changes in the perception of duration, rather than to biases in decision-making. Evidence for this difference came from the application of a drift-diffusion model (DDM) of perception and decision-making in which separable components are assigned to the perceptual accumulation of evidence or decision variables (Voss, et al., 2004).

The cue combination framework proposes a manner in which noisy estimates are combined optimally by shifting the temporal estimates toward their more precise input (Ernst and Banks, 2002; Alais and Burr, 2019); therefore, since movements have been shown to be precise, with low variability and high temporal fidelity, the variance of movement time estimates will also be low (Doumas et al., 2008; Brenner et al., 2012). However, the brain also integrates statistics of body movements such as the speed, length, direction, and area of movement with other sensory inputs (Petzschner et al., 2015) leading to biases in the perception of time. Previous studies have also demonstrated a “modality-appropriateness” effect in time perception in which time estimates are “pulled” toward the modality with the lower variance (van Wassenhove et al., 2008).

The specifics of Bayesian cue combination are that sensory estimates of time (*t*_S_) are drawn from a normal distribution, $t_{S}∼N(\mu ,\;\sigma )$, with a specific mean and variance, depending on the fidelity of the sensory modality conveying the temporal information. When two sensory estimates are available, each conveying a measure of duration, both are combined into a multisensory estimate. Assuming one signal is from a sensory (S) modality (e.g., auditory, visual) and the other is from movement (M), each with its own mean and variance, the combined sensorimotor (SM) estimate is given as follows:

$$\mu_{\mathrm{SM}}=w_{S}\times \mu_{S}+w_{M}\times \mu_{M},$$

wherein each independent channel is weighted. In this way, the peak of the SM distribution will be closer to whichever sensory estimate has the greater weight. The weights themselves for each estimate are then calculated (for M, as example) as follows:

$$w_{M}=\frac{\sigma_{S}^{2}}{\sigma_{M}^{2}+\sigma_{S}^{2}}.$$

That is, the weight for a given estimate is the ratio of the other modality's variance over the sum of the variances of both modalities. For the variance of the combined SM estimate, this can be expressed as the product of the other variances divided by their sum or as follows:

$$\sigma_{\mathrm{SM}}^{2}=\frac{\sigma_{S}^{2}\times \sigma_{M}^{2}}{\sigma_{S}^{2}+\sigma_{M}^{2}}.$$

**Figure 1.**
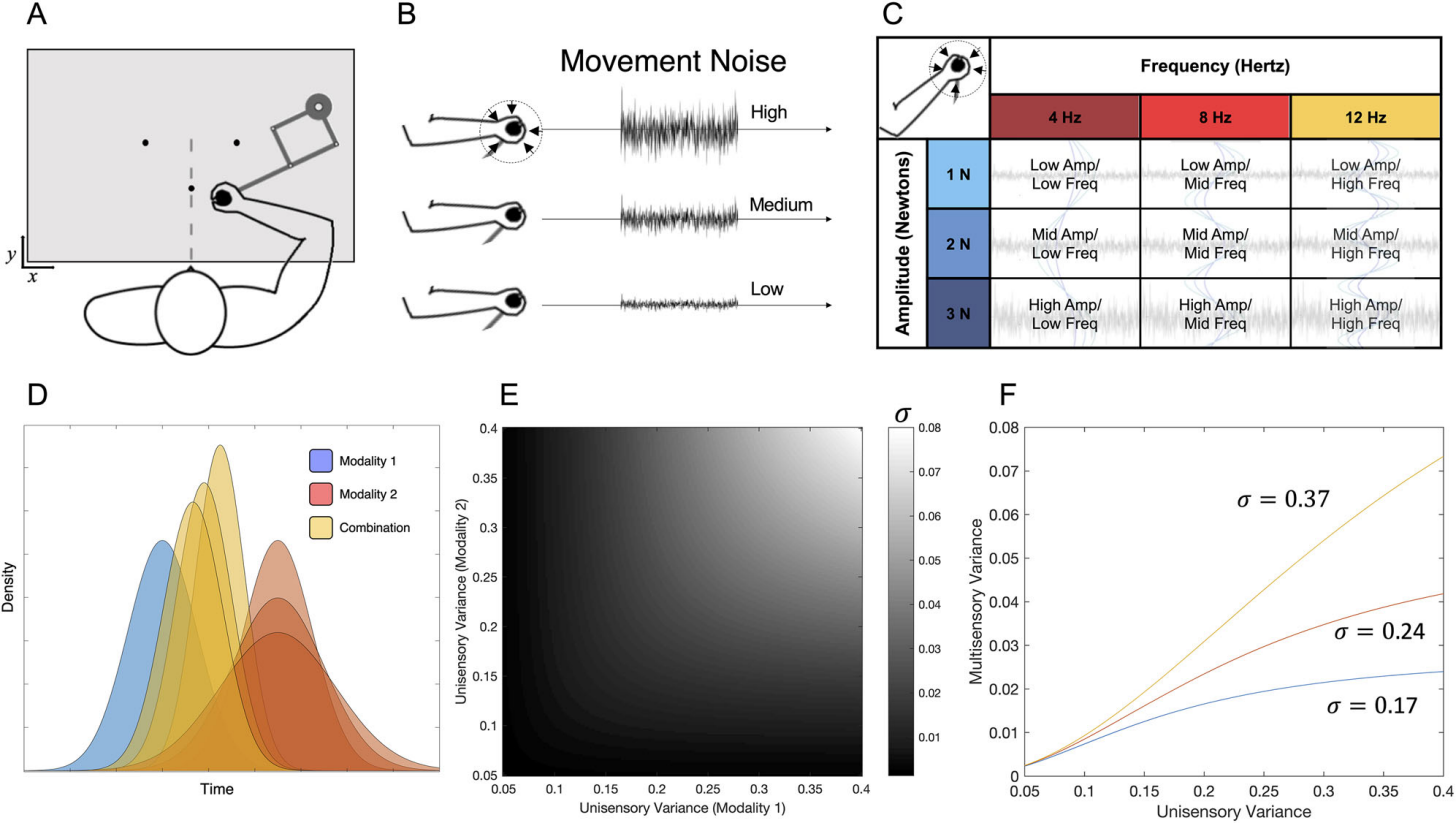
Experimental setup and cue combination model. ***A***, Subjects sat facing and held a robotic arm manipulandum that was kept below an occluding viewscreen. The experimental paradigm required subjects to begin each trial by moving the arm to a central starting location. On a given trial, subjects were required to categorize time intervals into “short” and “long” duration categories by moving the arm cursor to one of the two response locations, counterbalanced between subjects. ***B***, On any given trial, subjects experienced movement noise in the robotic arm, generated by introducing a tremor on the arm itself, in a random direction between trials, across one of the three noise levels (low, medium, high). ***C***, Movement noise was designed across three levels of two dimensions: the amplitude of the noise, expressed in Newton, or the frequency of the noise, expressed in frequency. ***D***, Bayesian cue combination predictions, in which two signals from separate modalities are combined optimally to form a combined distribution that is more precise than either distribution alone. If one modality is made less precise (wider width), the combined distribution also lowers in precision while becoming weighted progressively in location toward the more precise modality. ***E***, Multisensory combination widths, expressed as a function of the precision of both unisensory widths, provide predictions of precision as a function of either increasing or decreasing levels of noise. ***F***, When the precision of one modality is kept constant, increasing the variance of the other distribution generates variance curves that depend on the base precision of the first modality. Three levels of base precision are presented here (*σ = 0.17, 0.24, 0.37); with less precise base modalities, increasing noise in the second modality has progressively larger effects.

The essential result of this combination is that the SM estimate will always be more precise than either of the unisensory estimates, alone. By calculating these equations across a range of values, predictions can be made of the behavior of multisensory estimates across a wide range of possible variances (Fig. 1*D*–*F*). We note that the sensory estimates in these cases rely on an estimate of duration, which is progressively determined over time, rather than a quantity encountered immediately (e.g., pitch, brightness, size). The mechanisms for measuring duration, such as accumulator or state-space trajectories, have been described elsewhere (Paton and Buonomano, 2018; Tsao, et al., 2022) and are agnostic to the above calculations; that is, regardless of how time is estimated, the question is what the brain does when two separate estimates have been calculated by distinct sensory modalities.

Two predictions made by these equations are that (1) SM estimates of duration will be combined optimally and (2) the SM estimate will depend on the precision of both modalities. Recently, we tested the first prediction in a study in which subjects measured either a sensory (auditory) time interval or timed their own movements or both. We found that subjects’ perception of either auditory tones or their own movements were indistinguishable in terms of their precision, yet each produced systematic errors, with subjects overestimating auditory tone intervals and underestimating the duration of their own movements. Notably, the combined estimate was in between both and also the most accurate. However, despite this improvement, subjects exhibited a suboptimal combination of both modalities; yet, the degree of suboptimality depended on the individual's overall level of precision, with more precise subjects closer to the optimal estimate (De Kock et al., 2023). To clarify, a suboptimal combination in this case would be one in which the observed variance in time estimates is greater than the variance predicted by the cue combination equations.

For the second prediction of the cue combination framework, when a more precise modality (i.e., movement) becomes less reliable, or “noisy,” its influence on the estimate will decrease; therefore, if movements are made unreliably or with uncertainty, they should have less of an influence than other modalities on time estimates (De Kock et al., 2021a). Furthermore, the size of this effect should vary with the baseline level of precision of the sensory modality. That is, if the sensory estimate of duration is already precise, then increasing noise in movements will have less of an effect (Fig. 1*F*). Conversely, if the sensory estimate is already noisy, increasing noise in movements will have a larger effect.

How can noise be introduced into motor movements? Critically, we conceive of a noisier movement as one that is less reliable. In other words, one where subjects feel their movements cannot be trusted or depended upon. A common example of motor noise is the experience of tremor, which in clinical cases (e.g., Parkinson's disease and essential tremor) can severely disrupt motor control (McAuley and Marsden, 2000). In the present study, we applied a tremor to healthy participants making arm movements to test whether this source of noise disrupted timing performance. Participants used a robotic arm to perform a temporal categorization task while experiencing variable tremor frequencies and amplitudes. We hypothesized that this source of motor noise would disrupt timing precision, but not accuracy, as predicted by the Bayesian cue combination framework. That is, it is possible for subjects to be inaccurate in their estimates yet consistent in the estimates made (i.e., a high level of precision).

## Materials and Methods

### Participants

A total of 48 participants that were right-hand dominant with normal or corrected-to-normal vision completed the following two experiments (Experiment 1: 24 participants, 15 females; 9 males; mean age, 23 years old; Experiment 2: 24 participants, 18 females; 6 males; mean age, 21 years old) from the University of California (UC) Davis student population and surrounding area. All participants were screened for handedness using the Edinburgh handedness survey (Oldfield, 1971) and provided consent as approved by UC Davis Institutional Review Board.

### Apparatus

Participants completed both experiments using a robotic manipulandum (KINARM End-Point Lab, BKIN Technologies). They were seated in an adjustable chair in front of the manipulandum at the height at which their forehead could rest comfortably on the apparatus’ headrest. The horizontal display was mirrored from the downward facing LCD monitor positioned above which occluded the participant's view of most of their arm in order to reduce feedback of arm and hand position. Participants gripped the right handle of the apparatus and made movements within the screens perimeter and reaching movements to circular targets 0.5 cm in diameter, placed 14 cm apart on the sagittal axis of the body. Each target represented short and long responses counterbalanced across participants. The manipulandum continuously measured handle position, velocity, and force applied at a sampling rate of 1,000 Hz.

### Procedures

#### Experiment 1: auditory

Participants completed a temporal bisection task in which participants would move to the central target location where the manipulandum would lock in place. After 1,000 ms, the warm-up phase would begin by the handle being released and the words “Get Ready” being displayed on the screen. At this time, participants were told to move freely within the perimeter of the workspace. While participants moved, a tremor was mechanically induced by the manipulandum at one of the three amplitudes (1, 2, 3 N) and frequencies (4, 8, 12 Hz), the direction of which was randomly selected from 180° on each trial (note that the tremor rattled in both directions). Following a 2,000 ms delay, a 440 Hz tone sounded for one of the seven durations between 1,000 and 4,000 ms (1,000, 1,260, 1,580, 2,000, 2,520, 3,170, 4,000 ms). When the tone stopped sounding, the participants were to move to one of the two response circles as quickly and accurately as possible to categorize the auditory tone as a short or long duration compared with all tones experienced so far (reference-free categorization). If a response was made prior to the auditory tone ending, or if subjects stopped moving, the trial was discarded, and they were required to re-do the trial. The two targets were located at 105 and 75° equidistant from the starting location with response assignment (short or long) counterbalanced between participants. A total of 378 trials were run per session. Subjects were not given any explicit instructions regarding their movements or strategy, simply being allowed to move freely through the workspace.

#### Experiment 2: visual

The goal of Experiment 2 was to test the impact of induced tremor on visual interval timing. The task parameters were identical to the auditory task, except that participants were required to categorize visual intervals presented as a change in the background luminance of the manipulandum display screen from black to gray (RGB values of 64, 64, 64; hue, 160; luminance, 60). After the screen reverted to black, subjects indicated whether the durations were “short” or “long” by reaching to the appropriate target. We tested visual intervals using this global background change rather than a fixation target to avoid spatiotemporal processing effects unrelated to duration encoding. A total of 378 trials again were run per session.

### Analysis

In Experiments 1 and 2, movement distance and force measures were taken for each trial. Movement distance was defined as the summed distance traveled (point-by-point Euclidean distance between each millisecond time frame) during the stimulus tone. Force was similarly defined as the summed instantaneous force during the stimulus tone. In Experiment 1, reaction time (RT) was defined as the time elapsed between the tone offset and reaching one of the two choice targets, whereas in Experiment 2, it was the time between the luminance offset and target. Outlier trials were excluded for RT values greater than three standard deviations away from the mean of a participant's log-transformed RT distribution (Ratcliff, 1993). Additionally, we removed all trials with RTs below 200 ms or above 2,000 ms, in order to avoid issues with model fitting. For each participant, we plotted duration by average proportion of “long” responses. From here, we used the psignifit 4.0 software package to estimate individual BP and coefficients of variation (CV) for all frequency and amplitude values (Schütt et al., 2016); all curves were fit with a cumulative Gumbel distribution to account for the log-spaced nature of tested intervals (Wiener et al., 2019; De Kock et al., 2021). The BP was defined as the 0.5 probability point on the psychometric function for categorizing intervals as “long”; the CV was defined as half the difference between 0.75 and 0.25 points on the function divided by the BP.

### Computational modeling

To better dissect the results of Experiment 1, we decomposed choice and RT data using a DDM (Ratcliff, 1978; Wiecki et al., 2013; De Kock et al., 2021b). Due to the low number of trials available per condition, we opted to use hierarchical DDM (HDDM) as employed by the HDDM package (version 0.9.8) for Python (https://github.com/hddm-devs/hddm). In this package, individual subjects are pooled into a single aggregate, which is used to derive fitted parameters by repetitive sampling from a hypothetical posterior distribution via Markov chain Monte Carlo (MCMC) sampling. From here, the mean overall parameters are used to constrain estimates of individual-subject estimates. HDDM has been demonstrated as effective as recovering parameters from experiments with a low number of trials (Wiecki et al., 2013).

A recent extension to the HDDM package, the likelihood approximation network (LAN) module, allows for the “base” DDM to accommodate a wider variety of models (Fengler et al., 2021). Traditionally, the DDM consists of four parameters: the threshold difference for evidence accumulation (*a*), the drift rate toward each boundary (*v*), the starting point or bias toward a particular boundary (*z*), and the nondecision time (*t*), accounting for remaining variance due to nonspecific processes (e.g., perceptual, motor latencies). With the LAN module, additional parameters can be accessed and adjusted in model construction. For our purposes, we chose the so-called “Lévy Flight” model extension, in which the noise in momentary evidence accumulation is modified by a parameter (alpha) which interpolates between a Gaussian distribution and a Cauchy distribution. Recent work has shown that the Lévy Flight model can accommodate many instances of two-choice decision-making, with the additional feature that it can account for randomness in choice (Voss et al., 2019). We chose to use this model here, as the alpha parameter allows for another possible source of noise in the perceptual process.

Our initial model construction began by fitting the data from Experiment 1 using a so-called “full” DDM, as done in our previous work (De Kock et al., 2021b). In this model, the only condition by which parameters vary is the duration presented on each trial; all parameters were set to vary. We chose this model setup to replicate both our previous work with this model, as well as other work on time categorization tasks demonstrating. Model construction was conducted using the HDDMnnStimCoding class. Model sampling was conducted using 10,000 MCMC samples, with a burn-in of 1,000 samples and a thinning (retention) of every fifth sample. Individual model fits were assessed by visual inspection of the chains and the MC_err statistic; all chains exhibited low autocorrelation levels and symmetrical traces. The resulting model contains seven values for each of the four parameters, reflecting each of the seven durations tested.

Once the model fits were obtained for each duration, we next proceeded to model simulation, so as to demonstrate how changing our three hypothetical parameters (*v*, *a*, alpha) could influence precision and RT. To do this, we used the simulator_stimcoding class to generate data (1,400,000 trials each) from three separate models, with three levels within each model. The levels for each model were conducted by taking the parameter of interest for that model (*v*, *a*, or alpha) and multiplying them by 0.75 for each level; for example, if the drift rate for a given duration was 2, the next level would be 1.5, and the next would be 1.125.

Fits to the behavioral data to assess the effect of frequency and amplitude were conducted by creating a model in which all five parameters (*v*, *a*, *t*, *z*, alpha) varied according to the three levels of each (1, 2, 3 N for amplitude; 4, 8, 12 Hz for frequency). Model fitting used the same sampling method as described above, and again chain stability was assessed. We note that we attempted a similar modeling approach for Experiment 2 but found large instability and autocorrelations in the chains. This likely reflects the larger variability in Experiment 2 across all conditions.

## Results

### Experiment 1: auditory categorization

To begin, our first experiment tested 24 individuals on an auditory temporal categorization task (also referred to as temporal bisection), in a manner similar to our previous work (Wiener et al., 2019, De Kock et al., 2021b). Specifically, human participants sat facing forward while holding a robotic arm manipulandum under an occluding viewscreen (Fig. 1*A*). On a given trial, an auditory tone was played for one of seven possible intervals between one and four seconds (log-spaced). Subjects were required to move the cursor indicating arm position to one of the two response locations, equidistant from the starting location on a given trial. Crucially, we introduced three levels of movement “noise” while subjects moved the robotic arm, expressed as a tremor applied to the handle (Fig. 1*B*). We characterized noise across two different dimensions, in which both the amplitude of the tremor (1–3 N) and its frequency (4–12 Hz) could vary from trial to trial (Fig. 1*C*). Our decision to parametrically vary tremors across these two dimensions was driven by our agnostic view to what the motor system may consider “noisiness.” Additionally, the direction of the tremor varied randomly between trials. Similar to our previous studies, subjects were required to categorize the interval as quickly but as accurately as possible and only enter the response location once the auditory tone had completed; furthermore, subjects were required to maintain movement throughout the trial, with any violation leading to trial termination (see Extended Data Figure 2-2 for example trajectories).

**Figure 2.**
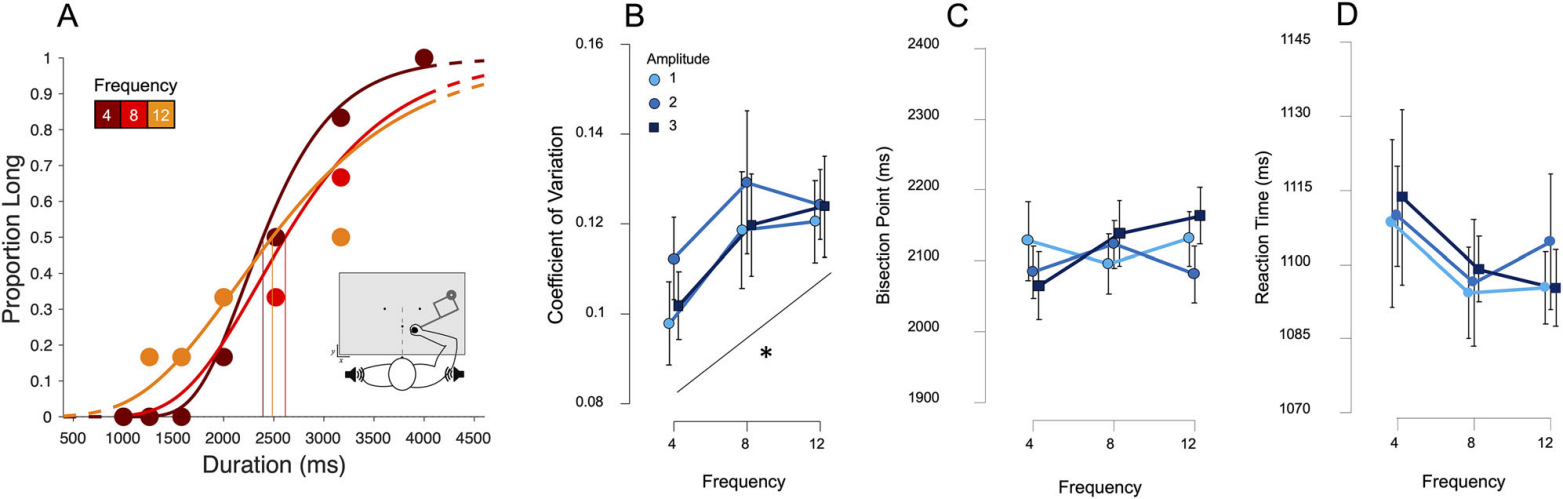
Results from Experiment 1 (auditory categorization). ***A***, psychometric curves fit to response proportions for a representative subject exhibiting a decrease in precision with increasing motor noise frequency, collapsed across amplitude; vertical lines indicate the BP (0.5 probability of classifying “long”). ***B***, Average CV for all subjects across all noise amplitudes and frequencies, demonstrating an increase for frequency, with no change for different amplitudes. ***C***, ***D***, Average BPs and reaction times across all conditions demonstrating no significant differences across either noise type. Error bars represent standard error; asterisk represents a linear effect at *p* < 0.05. See Extended Data Figures 2-2 and 2-3 for additional results on movement kinematics and example trajectories.

Analysis of choice and RT data also proceeded similarly to our previous reports, with choice data fit with a psychometric curve to calculate the bisection point (BP; 0.5 probability of choosing “long”) and the CV (half difference between 0.75 and 0.25 probability points divided by the BP); the BP reflects the level of bias in categorization, whereas the CV reflects the precision (Fig. 2*A*). A repeated-measure ANOVA revealed that there were no significant effects of amplitude (*F*_(2,46) _= 0.05; *p *= 0.955) or frequency (*F*_(2,46) _= 1.68; *p *= 0.197) nor was there an interaction effect of the two (*F*_(4,92) _= 1.42; *p *= 0.233) on BP (Fig. 2*C*). Therefore, participants were not distracted by the induced tremor and were able to accurately complete the task (Fig. 2). There were also no significant effects of frequency (*F*_(2,46) _= 1.37; *p *= 0.264), amplitude (*F*_(2,46) _= 0.13; *p *= 0.879), nor an interaction effect of frequency and amplitude (*F*_(4,92) _= 0.08; *p *= 0.988;) on RT (Fig. 2*D*).

10.1523/ENEURO.0013-24.2024.f2-1 Figure 2-1 Movement length and force effects across both experiments. Download Figure 2-1, TIF file.

10.1523/ENEURO.0013-24.2024.f2-2 Figure 2-2 Example trajectories for two sample subjects from Experiments 1 (top) and 2 (bottom). Each panel displays all of the trajectories for the middle duration trials (2000ms). Center black point represents the starting zone whereas the upper two locations are the target response zones. Like previous reports, subjects adopted idiosyncratic yet consistent movement strategies during the task. Download Figure 2-2, TIF file.

However, a repeated-measure ANOVA of the CV revealed a significant effect of frequency (*F*_(2,46) _= 3.85; *p *= 0.028; $\eta_{p}^{2}$ = 0.143); there was no significant effect of amplitude (*F*_(2,46) _= 0.46; *p *= 0.636) nor an interaction effect of amplitude and frequency (*F*_(4,92) _= 0.10; *p *= 0.983). A further examination revealed this to be a linear effect, with CV values generally increasing with frequency (*t *= 2.425; *p *= 0.019; Cohen's *D *= 0.715) suggesting precision decreased as frequency increased (Fig. 2*B*).

A control analysis on the direction of the movement tremor was also conducted, by dividing responses into three separate bins, depending on the direction of the tremor (bin 1, 0–60°; bin 2, 61–120°; bin 3, 121–180°). Here, no effect of tremor direction was detected for either the BP (*F*_(2,46) _= 0.321; *p = 0*.727) or the CV (*F*_(2,46) _= 0.755; *p *= 0.476), suggesting the direction of the tremor had no effect on either bias or precision.

In addition to the response variables, we also wanted to evaluate the parameters of movements made while encoding the duration, specifically movement length and force (Extended Data Figure 2-1). A repeated-measure ANOVA for movement length revealed no significant effect of frequency (*F*_(2,46) _= 2.93; *p *= 0.063) or amplitude (*F*_(2,46) _= 0.01; *p *= 0.994) nor an interaction effect (*F*_(4,92) _= 1.97; *p *= 0.106); although the frequency effect is not significant, the data and our hypotheses suggest a linear effect of frequency but not amplitude on movement length; therefore, we explored the linear comparison contrast of frequency across all levels of amplitude which revealed a significant effect (*t *= −2.24; *p *< 0.05). As expected from previous research (Wiener et al., 2019; De Kock et al., 2021b), movement length increased with duration (*F*_(6,138) _= 254.07; *p *< 0.001).

A repeated-measure ANOVA of movement force revealed a significant effect of frequency (*F*_(2,46) _= 34.41; *p *< 0.001), amplitude (*F*_(2,46) _= 5.26; *p *< 0.05), and an interaction effect of frequency and amplitude (*F*_(4,92) _= 9.44; *p *< 0.001). Post hoc analysis of frequency showed that movement force was not significantly higher at the lowest frequency (Freq4) compared with the mid frequency (Freq8; *t *= 2.36; *p *= 0.081) and significantly higher compared with the highest frequency (Freq12; *t *= 8.17; *p *< 0.001). Movement force was also significantly higher at the mid frequency (Freq8) compared with the highest frequency (Freq12; *t *= 5.28; *p *< 0.001). Post hoc analysis of amplitude showed that movement force was significantly lower at the lowest amplitude (Amp1) compared with the mid amplitude (Amp2; *t *= −3.70; *p *< 0.01) and the highest amplitude (Amp3; *t *= −2.94; *p *< 0.001). Movement force was also significantly lower at the mid amplitude (Amp2) compared with the highest amplitude (Amp3; *t *= −4.97; *p *< 0.001).

### Computational modeling

The results of Experiment 1 support, in principle, the predictions of the Bayesian cue combination model. That is, if two sensory modalities both convey estimates of time, these estimates will be combined optimally to improve perceived duration. However, if one of those modalities becomes noisier, and so less reliable, the overall precision will decrease as the noise increases (Hartcher-OBrien et al., 2014). Furthermore, the decrease in precision should diminish with increasing noise, depending on the precision of the unchanged modality. If movement represents a sensory channel for estimates of duration, then increasing noise in movements should decrease the precision of time estimates. This prediction was borne out in our data; however, alternative explanations to the findings exist. Indeed, decreases in timing precision (increases in CV) can be explained by numerous effects in the timekeeping process; noisier time estimates may result from a poorer ability to encode time or from an impairment in remembering those durations or also a difference in how those durations are judged (Allman et al., 2014). In our previous report, we had shown that increasing movement viscosity shifted response bias (changes in the BP) while sparing precision (De Kock et al., 2021b). There, we found through computational modeling of behavior that the effect likely arose from a difference in perception rather than a change in decision-making. We chose to take a similar approach here.

To begin, we opted to again employ a DDM framework. The classic DDM is able to account for a wide variety of effects in choice and RT by accounting for the shape of response distributions across different experimental levels (Ratcliff, 1978). The typical DDM assumes that information is accumulated over time in a noisy stochastic process toward one of the two response thresholds, with a given boundary separation. The rate of accumulation is determined by the drift rate parameter (*v*) and is corrupted by white noise on a moment-by-moment basis during the accumulation process, until the boundary is reached, given by the threshold parameter (*a*). Additional parameters include a delay in the initiation of accumulation, known as nondecision time (*t*) and starting-point bias toward one of the two boundaries (*z*).

Here, we used the HDDM (version 0.9) package for model construction and simulations (Wiecki et al., 2013). HDDM allows for the construction and fitting of hierarchical DDMs by constraining individual-level parameter estimates on the basis of group-level ones in addition to prior distributions for the given parameters. For the present study, we employed the recently updated LAN extension to HDDM, in which a wider number of possible models are now supported by training through artificial neural networks (Fengler et al., 2021). These model extensions include those with collapsing boundary or leak parameters. Of relevance to the present study, the LAN extension also includes a so-called Lévy Flight model (Voss et al., 2019). In this model, the noise for the momentary sensory evidence is modified by the parameter (alpha), which determines the shape of the noise distribution. This parameter, which ranges between 1 and 2, interpolates the shape of the noise distribution between a Gaussian distribution at higher values and a Cauchy distribution at lower values. The Cauchy distribution includes heavy tails in both directions and so allows for large “jumps” in evidence accumulation toward either of the decision boundaries. As the Cauchy is narrower overall than the Gaussian, the accumulation process may be less noisy moment-to-moment yet overall may shift randomly by a large amount.

**Figure 3.**
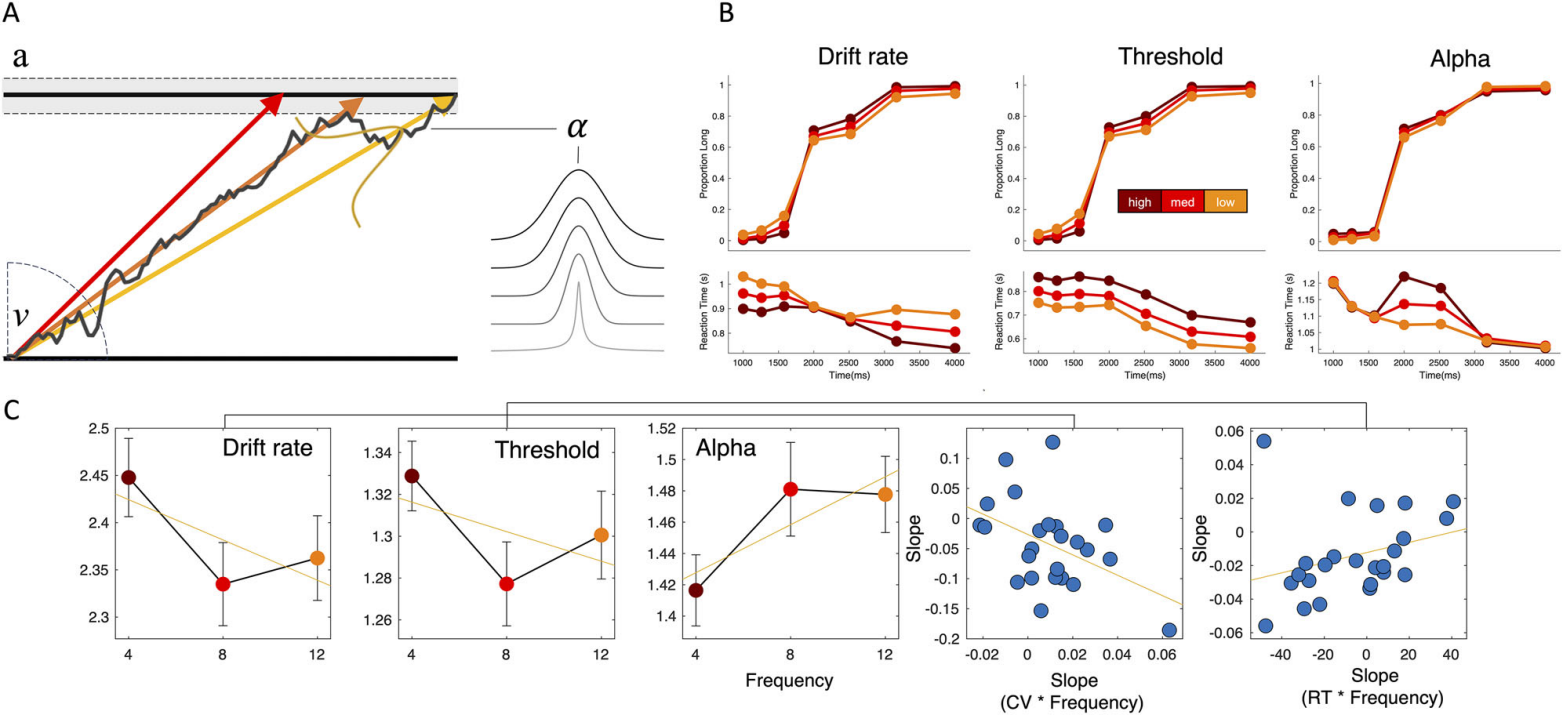
Computational modeling using a DDM framework with Lévy Flights. ***A***, DDM framework in which evidence is accumulated to a boundary for categorizing a time interval as long or short. The rate of accumulation on a given trial is determined by the drift parameter (v; colored lines represent three possible drift rates on different trials), while the boundary for the response is determined by the threshold parameter (a; dashed lines represent different possible levels on different trials); additionally, the momentary noise of the distribution is determined by the noise transformation parameter (alpha) which determines the relative shape of momentary noise, interpolating between a Gaussian distribution and a Cauchy distribution (heavy tailed) at higher and lower levels, respectively. ***B***, Choice and RT findings from simulated models with each of the three possible parameters varying over three different levels. All three models lead to changes in choice precision by increasing parameter values, but with different RT predictions. ***C***, Fits of the DDM–Lévy model, collapsed across amplitude, reveal significant changes in all three parameters with increasing movement noise frequency (left three panels). However, only the drift rate effect correlates with the CV effect of behavior, whereas the threshold effect correlates with changes in RT.

For our model simulations, we suggest that “noise” may be considered by changing either the drift rate, threshold, or alpha parameters (Fig. 3*A*). To accomplish this, we began by fitting a DDM–Lévy model to our full dataset, with all parameters included [*v*, *a*, *t*, *z*, alpha] but only duration as a conditional parameter. We chose to include these parameters on the basis of our previous work and others demonstrating these parameters (excluding alpha) as the best for accounting for behavior on this particular task and also to follow a priori assumptions. Once the fitted parameters were obtained, we next simulated three separate datasets using these same parameters but by varying each of the three parameters described above across three different levels (see Materials and Methods).

For the drift rate, when considered as an absolute value (i.e., the drift rate may be considered either signed, pointing toward one boundary or another, or unsigned, indicating overall slope), lower drift rates would indicate a slower accumulation process, which has been shown to relate to the overall signal-to-noise ratio in perception. We simulated three separate datasets with three drift rates (high, medium, low) and observed that as the drift rate lowers, the psychometric curves becomes less steep, indicating a decrease in precision. Notably, the RT shape across intervals also changes, with lower drift rates associated with longer RTs, but only for the longest and shortest interval in the stimulus set. For the threshold parameter, lowering the threshold also resulted in a decrease in precision, yet here the RT distribution shifted uniformly across all intervals, with responses becoming quicker for lower threshold. This effect is due to lower thresholds leading to faster responses, which require less evidence accumulation. For the alpha parameter, we again observed a decrease in precision but for lower values of alpha instead of higher values. That is, as the noise more closely approximated a Cauchy distribution, the shallower the psychometric curve became. Furthermore, RTs increased with lower alpha values but only for the middle durations; this effect likely results from a longer amount of time necessary with a more constrained evidence accumulation regime to reach a specified boundary, thus lengthening the time before a response is committed (Fig. 3*B*).

Altogether, all three models could provide an explanation for decreases in precision resulting from increases in noise, yet with differing predictions for RT. After fitting the full model to our data, we observed that all three parameters additionally shifted with changes in frequency (Fig. 3*C*). More specifically, we observed a decrease in drift rate as frequency increases (*F*_(2,46) _= 9.962; *p < *0.001), in addition to a decrease in threshold (*F*_(2,46) _= 14.661; *p *< 0.001) and an increase in alpha (*F*_(2,46) _= 4.98; *p = 0*.011). Across these parameters, we note that only the change in the drift rate and threshold were consistent with the model simulation results; an increase in alpha predicts better precision rather than a decrease as observed in our behavioral data. However, for the drift rate and threshold, either may match the behavioral data. To determine which, we calculated the slope of a linear regression across frequency for both the drift rate and threshold and correlated those values between-subject with the slope of the CV values across frequency. Here, we observed that only the drift rate effect significantly correlated with the CV effect (Pearson's *r *= −0.451; *p *= 0.026; Spearman's *r *= −0.413; *p *= 0.044), whereas the threshold effect did not (Pearson's *r *= −0.213; *p *= 0.317; Spearman's *r *= −0.281; *p *= 0.182). Conversely, we found that the threshold effect could explain changes in RT across frequency levels (Pearson's *r *= 0.296; *p *= 0.159; Spearman's *r *= 0.413; *p *= 0.044; we note the lack of a Pearson's effect here is likely driven by an outlier, for which the Spearman effect is not affected), whereas drift could not (Pearson's *r *= −0165; *p *= 0.438; Spearman's *r *= −0.086; *p *= 0.685). Although the RT effect was not significant, we note that theoretically the threshold parameter should be able to explain any between-subject differences in RT.

### Experiment 2: visual

The overall findings of Experiment 1 and computational modeling both support the notion that increasing the frequency of movement noise leads to a decrease in perceptual timing precision in accordance with Bayesian cue combination. However, recall in the cue combination framework that the overall effect of increasing noise on one modality will depend on the base level of precision in the other modality; if the unchanged modality's precision is high, then the impact of increasing noise in the second modality will diminish with higher levels of noise, whereas if the unchanged modality's precision is low, then increasing noise in the second modality will have a larger effect with less diminishment. To test this possibility, we repeated our temporal categorization task in a new sample of subjects (*n* = 24) but with the visual modality used for timing instead of auditory. It is well documented that the fidelity of perceptual timing is worse for visual than for auditory stimuli, such that precision is lower for the former than the latter (van Wassenhove et al., 2008; Shi et al., 2013; Wiener et al., 2014). In place of an auditory tone, the visual interval was demarcated by a global change in luminance of the viewscreen (see Materials and Methods). This was done so that subjects would be able to easily attend to the onset/offset of the stimulus regardless of where they were looking on the screen or where the cursor was located (see Extended Data Figure 2-2 for example trajectories).

**Figure 4.**
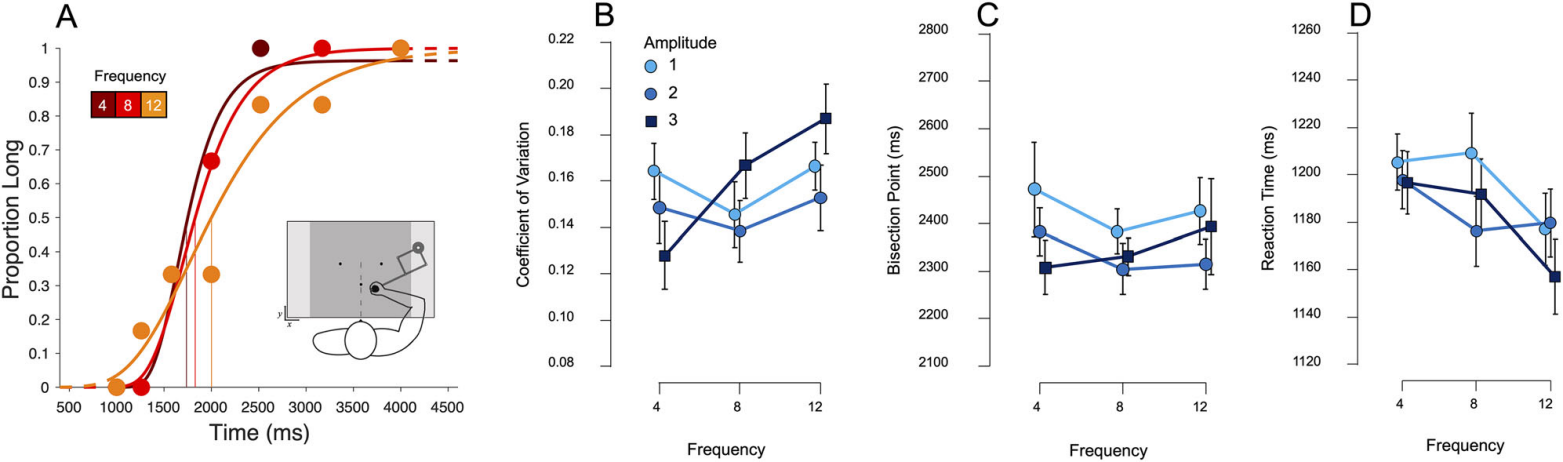
Results from Experiment 2 (visual categorization). ***A***, Psychometric curves fit to response proportions for a representative subject again exhibiting a decrease in precision with increasing movement noise frequency (highest amplitude). ***B***, Average CV for all subjects across all noise amplitudes and frequencies; in contrast to Experiment 1, an increase in CV across frequency was observed only for the highest amplitude noise (3 N). ***C***, ***D***, Average BPs and reaction times across all conditions again demonstrating no significant differences across either noise type. Error bars represent standard error. See Extended Data Figures 2-2 and 2-3 for additional results on movement kinematics and example trajectories.

As in Experiment 1, we also analyzed BP and CV as measure of bias and precision (Fig. 4*A*), respectively, using repeated-measure ANOVAs. Similar to those results, there was no effect of amplitude (*F*_(2,46) _= 2.23; *p *= 0.119) or frequency (*F*_(2,46) _= 0.42; *p *= 0.66) nor an interaction effect (*F*_(4,92) _= 0.39; *p = 0*.814) on BP, again suggesting participants were also not distracted by the noise or stimuli and were able to accurately complete the task (Fig. 4*C*). There was again no effect of frequency (*F*_(2,46) _= 2.28; *p *= 0.114), amplitude (*F*_(2,46) _= 0.83; *p *= 0.443), or an interaction effect (*F*_(4,92) _= 0.82; *p *= 0.515) on RT (Fig. 4*D*). A control analysis on tremor direction again was conducted on choice responses, which once again failed to find any effect on either the BP (*F*_(2,46) _= 0.067; *p = 0*.935) or the CV (*F*_(2,46) _= 0.455; *p = 0*.637).

For the CV, we again found no effect of amplitude (*F*_(2,46) _= 1.01; *p *= 0.374) or an interaction effect of amplitude and frequency (*F*_(4,92) _= 1.58; *p *= 0.188). However, we did not observe a main effect of frequency (*F*_(2,46) _= 2.61; *p *= 0.084; $\eta_{p}^{2}$ = 0.102); we note that the linear contrast was significant (*t*_(46) _= 2.121; *p *= 0.039; *D *= 0.625), indicating an overall increase in CV with higher frequencies. Following our a priori hypotheses, we analyzed simple main effects with frequency as the simple effect factor and amplitude as the moderator. Here, we found a significant effect of frequency only for the highest amplitude of three Newtons (*F*_(2,46) _= 3.844; *p *= 0.029), but not for either of the lower amplitudes (*p *= 0.414 and 0.766, respectively), indicating that the CV did significantly increase but only for the highest amplitude (Fig. 4*B*).

We again analyzed movement length and force in addition to the response variables (Extended Data Figure 2-1). A repeated-measure ANOVA of movement length showed no significant effect of amplitude (*F*_(2,46) _= 2.68; *p *= 0.079) and no significant effect of frequency (*F*_(2,46) _= 2.06; *p *= 0.139) nor an interaction effect (*F*_(4,92) _= 0.96; *p *= 0.431). Given the marginal effect of amplitude and the linear nature of the data, we decided to explore the linear comparison of amplitude averaged over all levels of frequency which revealed a significant effect (*t*_(46) _= 2.30; *p *< 0.05). As expected from previous research (Wiener et al., 2019; De Kock et al., 2021b) and Experiment 1 results, movement length increased with duration (*F*_(6,138) _= 309.25; *p *< 0.001).

A repeated-measure ANOVA on movement force, however, revealed a significant effect of amplitude (*F*_(2,46) _= 4.04; *p *< 0.001), frequency (*F*_(2,46) _= 43.20; *p *< 0.001), and an interaction effect (*F*_(4,92) _= 4.76; *p *< 0.001). Post hoc analysis of amplitude showed that movement force was significantly lower at the lowest amplitude (Amp1) compared with the mid amplitude (Amp2; *t *= −3.40; *p *< 0.01) and highest amplitude (Amp3; *t *= −5.50; *p *< 0.001). It was also significantly lower at the mid amplitude (Amp2) compared with the highest amplitude (Amp3; *t *= −4.97; *p *< 0.001). Post hoc analysis of frequency showed that movement force was significantly higher at the lowest frequency (Freq4) compared with the highest frequency (Freq12; *t *= 7.24; *p *< 0.001) and not significantly higher than the mid frequency (Freq8; *t *= 7.83; *p *= 0.081). Movement force was also significantly higher at the mid frequency (Freq8) compared with the highest frequency (Freq12; *t *= 7.83; *p *< 0.001).

### Cross-modal comparisons

In order to investigate differences between the two modalities (Exp 1., auditory; Exp 2., visual), we conducted a mixed-model ANOVA with modality as the between-subject factor. Although at first glance it appears that movement force was overall higher in the visual modality (Exp. 1) than in the auditory modality (Exp. 2), this difference was not significant (*F*_(1,46) _= 0.92; *p *= 0.341). Movement length was found to increase as frequency increased in the auditory modality (Exp. 1); however, in the visual modality (Exp. 2), movement length increased as amplitude increased. We again compared across modalities and found that movement length was not significantly longer in the visual modality (Exp. 2) compared with the auditory modality (Exp. 1; *F*_(1,46) _= 3.47; *p *= 0.069). Together these findings suggest that increases in the size of the tremor led to increased movement force and longer movement lengths, whereas increases in the speed of the tremor led to decreased movement force and so shorter movement lengths. Previous research has suggested that increases in movement length lead to increases in time perception (Wiener et al., 2019; De Kock et al., 2021b); therefore, a logical next step was to compare the BP across the two modalities to see if this was the case here as well. However, cross-modal analysis revealed no significant differences on BP between groups (*F*_(1,46) _= 2.83; *p *= 0.099). In addition to response variables and movement parameters, we also verified that there were no significant differences of modality on RT (*F*_(1,46) _= 1.48; *p *= 0.230). This finding further supports the suggestion that the effects of frequency and amplitude on the different movement parameters did not influence the perception of time but was simply due to the nature of controlling the robotic arm with the increasing size and speed of movements.

**Figure 5.**
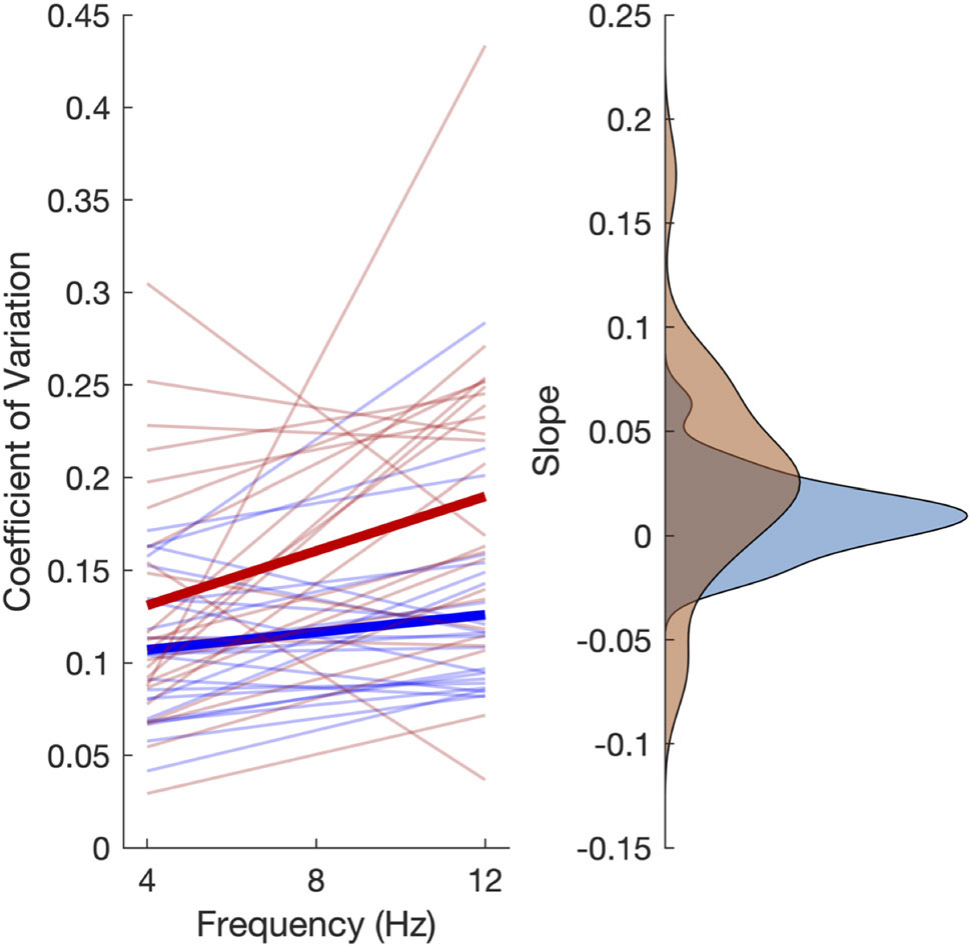
Slope comparisons between experimental groups. The left panel displays linear regressions of the CV for individual subjects and group averages (bold lines; red, visual; blue, auditory) across the three noise frequencies. The right panel displays density estimates of the distribution of slope values for each. We observed that subjects performing the task with visual stimuli, in addition to higher CV values overall, exhibited a larger influence of noise frequency, as exhibited by a significantly higher slope value for these subjects.

Lastly, and most important, was the cross-modal comparison of CV which revealed precision was overall lower (higher CV values) in the visual modality compared with the auditory modality (*F*_(1,46) _= 10.01; *p *< 0.01; $\eta_{p}^{2}$ = 0.179). Additionally, we evaluated the size of the effect of frequency on each modality as, according to the cue combination framework, the influence of noise on modality should depend on the base precision of the unchanging modality, and so if the visual modality is less precise, then noise should have a larger effect. To test this, we calculated slope values for each effect and compared them. For the auditory group, we used the average effect of frequency, collapsed across amplitude, whereas for the visual group, we only used the effect of frequency for the highest amplitude (Fig. 5). Here, a significant difference was found, with the effect of noise having a larger influence for the visual group than the auditory one (Mann Whitney *U* = 170; *p = 0*.014; rank biserial correlation = −0.41).

## Discussion

The overall purpose of these experiments was to test the proposed Bayesian cue combination framework which suggests that movement serves as an additional channel of temporal information that has high precision and low temporal fidelity which improves the precision of time perception. We therefore considered, if movement became unreliable or noisy, we should see a decrease in the precision of timing in that, it will be pulled toward the more precise sensory input. We additionally wanted to investigate whether this effect would differ between auditory and visual stimulus. Given previous findings that auditory temporal perception is more precise than visual (Wiener et al., 2014; Mioni et al., 2016), we hypothesized that noisy movements would lead to less precise estimations of time with visual stimuli compared with auditory.

Different levels of amplitude or frequency did not have a significant effect on accuracy in either the auditory or visual version of the study suggesting that participants were able to appropriately complete the task and the noise did not serve as a distractor from the timing task. This distinction is important, as one might expect that increased tremors would lead subjects to pay less attention to the timing task. However, in that case, the prediction is that the time estimates themselves would also become more biased to be shorter (Brown, 1997; Fortin, 2003), which was not the case. There were also no significant effects of frequency or amplitude on RT within or between either experiments.

We did find an effect of precision in that an increase in frequency but not amplitude led to a less precise perception of time for auditory stimuli, whereas for visual stimuli, the effect of frequency was only found at the highest level of amplitude. Specifically, for Experiment 1 (auditory), participants were most precise at the lowest frequency level (4 Hz) with a leveling off between the mid (8 Hz) and high frequency (12 Hz). For Experiment 2 (visual), we found the same pattern but only for the highest amplitude (3 N), whereas for low (1 N) and mid (2 N) amplitudes, there were no significant differences in precision. Therefore, when participants timed auditory tones, increasing the speed of the tremor but not the size of the tremor caused a decrease in precision. One explanation for this difference is the difference in baseline precision between auditory and visual modalities; that is, since auditory time estimates are already very precise, small disruptions to movements will lead to a change in their overall precision. In contrast, since visual time estimates are already less precise, a larger amount of noise is necessary in movements to induce an effect, as the noise in movements must exceed the noise of visual time estimates such that the cue combination begins to favor them over movements. As such, larger noise is only achieved in the visual experiment at a high amplitude.

The computational modeling conducted in our study allowed us to identify possible sources of noise in the perceptual process. By relying on a DDM framework that incorporates Lévy Flights, we observed that the behavioral effect could be explained by a shallower drift rate. Decreases in drift rate have been associated with a lower signal-to-noise ratio and so relate to the rate of evidence accumulation in the perceptual process (Voss et al., 2004; Palmer et al., 2005; Rohenkohl et al., 2012). In our case, when the drift rate was lower, we observed a similar finding to our behavioral data. Furthermore, fits of this model to the behavioral data revealed that changes in the drift rate correlated with changes in precision, supporting the conclusion that the effects were driven by perception-level changes rather than biases in decision-making or changes in strategy.

As for movement parameters during temporal encoding, in the auditory version (Exp. 1), we found there to be a linear effect of frequency but not amplitude on movement length; specifically, movement length significantly decreased linearly with an increase in frequency for the two higher amplitude levels (Amp2 and Amp3) but not at the lowest amplitude (Amp1). Notably, for the visual version of the study (Exp. 2), there was a linear effect of amplitude but not frequency where movement length significantly increased linearly with an increase in amplitude for the lowest frequency (Freq4).

We also observed differences in movement length between experiments in that movement length was overall longer in the visual study (Exp. 2) compared with the auditory study (Exp. 1); however, it was only marginally significantly different. Given previous findings and in line with proposed framework, we would expect a difference in movement length would lead to a biasing effect of time perception; however, there was no a significant difference between the two experiments on BP which suggests that movement length did not influence time perception in this case.

The results of our experiment have particular implications for the study of movement disorders. Notably, time perception abilities are impaired in pathologies of movement, such as Parkinson's disease (Singh et al., 2021), Huntington's disease (Lemoine et al., 2021), or cerebellar degeneration (Breska and Ivry, 2021) but also in essential tremor (Pedrosa et al., 2016), Tourette's syndrome (Vicario et al., 2010), and dystonia (Conte et al., 2017). Conversely, individuals with highly trained coordination, such as professional athletes or musicians, exhibit enhanced timing abilities (Cicchini et al., 2012; Chen et al., 2016). Our observation that introducing a tremor to otherwise healthy individuals disrupts perception suggests an intrinsic link between motor symptomatology and perceptual processes. This link may further go beyond perception into the cognitive domain as well. Indeed, work with subjects with attention-deficit/hyperactivity disorder, where well-known timing disruptions exist (Smith et al., 2002), has shown that ancillary movements can lead to improvements in perceptual processes (Hartanto et al., 2016). Similarly, recent work in Parkinson's patients has linked deficits in SM timing to cognitive impairments (Singh et al., 2021). A corollary, implied by the present findings, is that by improving motor symptoms one could also improve both perception and cognition. Therefore, one might suggest that motor rehabilitation can also lead to other benefits in these patients. In the case of timing, it is possible to improve SM estimates through repeated training, which can lead to functional and morphological changes in SM brain regions (Bueti et al., 2012), yet whether this also improves other symptoms is unknown.

A second future avenue of research relates to the frequencies employed in the present study. Here, we chose the specific range (4–12 Hz) to reflect that observed in motor system tremors (McAuley and Marsden, 2000). We note, however, that the higher end of the tremors is closer to that identified as the so-called SM “mu” rhythm (8–13 Hz; Pineda, 2005). While traditionally involved in movements, recent work has linked mu oscillations to motor-related changes in timekeeping processes, both for single intervals and rhythmic ones (Iwasaki et al., 2018; Ross et al., 2022). Notably, mu rhythms exhibit suppression during action initiation. One possibility, then, is the effects observed in the present study relate to a kind of resonance with mu oscillations via the induced tremor, thus leading to the observed disruption. Neural recordings, combined with a wider array of tremor frequencies, could shed light into this possibility, which would provide a distinct mechanism by which tremors exert their influence on the motor system.

Overall, our method, which relies on a novel use of a robotic arm to mimic tremors, was able to effectively alter timing performance. We suggest that our findings are not due to changes in attention or decision-making but instead result from a fundamental change in perceptual processing, which follows from the Bayesian cue combination account. In conjunction with our other findings, we now find converging evidence to support the cue combination account for how movements influence perceptual time estimates, which in turn supports the hypothesis that movements themselves act as a timekeeping process with high fidelity.

## Synthesis

Reviewing Editor: James Howard, Brandeis University Decisions are customarily a result of the Reviewing Editor and the peer reviewers coming together and discussing their recommendations until a consensus is reached. When revisions are invited, a fact-based synthesis statement explaining their decision and outlining what is needed to prepare a revision will be listed below. The following reviewer(s) agreed to reveal their identity: Ramesh Balasubramaniam, David Robbe.

Main Comments

1. Although the study emphasizes the importance of movement, the authors do not show the movement trajectories performed by the participants. Participants could decide on whether the interval is short or long by moving first toward the short target and then the long one. It may not have been possible to capture the movement data in the current study, but if it was captured could the authors show the data or explain in the main text why they didn't show it?

2. There is a very limited number of citations given in the introduction regarding the importance of movement in time estimation. There are several interesting reviews that dealt with the role of movement and sensorimotor dynamics in explicit time perception (e.g., Coull and Droit-Volet, 2018). Robbe (2023) also recently provided a comprehensive cross-species overview of behavioral and neurophysiological evidence suggesting time estimation is intrinsically dependent on movement. Similarly, when the authors wrote at the end of their manuscript that their data "which, in turn, supports the hypothesis that movements themselves act as a timekeeping process with high fidelity," it would be nice to cite studies or reviews that have actually made that claim.

3. The following statement is repeated at several places in the manuscript: "When two sensory estimates are available, each conveying a measure of duration, both are combined into a multisensory estimate." Can the authors explicitly explain how a constant tone or a visual stimulus provides a measure of duration? It's understandable how a regular movement can provide information about time (distance traveled will increase with time), but if the tone is sustained, it provides absolutely no temporal information, correct?

4. The authors should address why there is not a more predictable monotonic patter in Figure 6. Do the authors have any specific reason why the middle frequency tremor condition behaves differently? Commenting on this could add some insight into the mechanisms involved.

Minor:

1. Were instructions given to the participants on how to move? A description of these instructions seems to be missing from the methods section.

2. The authors should clarify the following statement: "We hypothesized that this source of motor noise would disrupt timing precision, but not accuracy." This seems to suggest that one could have a precise but inaccurate estimate, but the authors should explicitly define those two terms.

3. Regarding the following statement: "Further, this effect was tied to changes in the perception of duration rather than to biases in decision making." The authors should clarify how perception and decision making can be clearly separated.

4. At several places, the authors mentioned that two estimates should be combined optimally. The authors should explain why this must be the case, and what are the criteria to define optimal versus non-optimal combination.

5. Regarding the following statement: "But in such a way that higher levels of noise saturated the impact, consistent with optimal integration." This statement is far from easily understandable. The authors should unpack such a statement and explain why saturation is consistent with optimal integration.

## References

[B1] Alais D, Burr D (2019) Cue combination within a Bayesian framework. In: *Multisensory processes* (Lee AKC, Wallace MT, Coffin AB, Popper AN, Fay RR, eds), pp 9–31. Cham, Switzerland: Springer International Publishing.

[B2] Allman MJ, Teki S, Griffiths TD, Meck WH (2014) Properties of the internal clock: first- and second-order principles of subjective time. Annu Rev Psychol 65:743–771. 10.1146/annurev-psych-010213-115117 24050187

[B3] Brenner E, van Dam M, Berkhout S, Smeets JBJ (2012) Timing the moment of impact in fast human movements. Acta Psychol 141:104–111. 10.1016/j.actpsy.2012.07.002 22864313

[B4] Breska A, Ivry RB (2021) The human cerebellum is essential for modulating perceptual sensitivity based on temporal expectations. Elife 10:e66743. 10.7554/eLife.66743 34165079 PMC8245126

[B5] Brown SW (1997) Attentional resources in timing: interference effects in concurrent temporal and nontemporal working memory tasks. Percept Psychophys 59:1118–1140. 10.3758/BF03205526 9360484

[B6] Bueti D, Lasaponara S, Cercignani M, Macaluso E (2012) Learning about time: plastic changes and interindividual brain differences. Neuron 75:725–737. 10.1016/j.neuron.2012.07.019 22920262

[B7] Chen YH, Verdinelli I, Cesari P (2016) Elite athletes refine their internal clocks: a Bayesian analysis. Motor Control 20:255–265. 10.1123/mc.2014-0070 26061186

[B8] Cicchini GM, Arrighi R, Cecchetti L, Giusti M, Burr DC (2012) Optimal encoding of interval timing in expert percussionists. J Neurosci 32:1056–1060. 10.1523/JNEUROSCI.3411-11.2012 22262903 PMC6621155

[B9] Conte A, McGovern EM, Narasimham S, Beck R, Killian O, O’Riordan S, Reilly RB, Hutchinson M (2017) Temporal discrimination: mechanisms and relevance to adult-onset dystonia. Front Neurol 8:625. 10.3389/fneur.2017.00625 29234300 PMC5712317

[B10] Coull JT, Droit-Volet S (2018) Explicit understanding of duration develops implicitly through action. Trends Cogn Sci 22:923–937. 10.1016/j.tics.2018.07.011 30266151

[B11] De Kock R, Gladhill KA, Ali MN, Joiner WM, Wiener M (2021a) How movements shape the perception of time. Trends Cogn Sci 25:950–963. 10.1016/j.tics.2021.08.002 34531138 PMC9991018

[B13] De Kock R, Zhou W, Joiner WM, Wiener M (2021b) Slowing the body slows down time perception. Elife 10:e63607. 10.7554/eLife.63607 33830016 PMC8051945

[B12] De Kock R, Zhou W, Datta P, Mychal JW, Wiener M (2023) The role of consciously timed movements in shaping and improving auditory timing. Proc Biol Sci 290:20222060. 10.1098/rspb.2022.2060 36722075 PMC9890119

[B14] Doumas M, Wing AM, Wood K (2008) Interval timing and trajectory in unequal amplitude movements. Exp Brain Res 189:49–60. 10.1007/s00221-008-1397-6 18483723

[B15] Ernst MO, Banks MS (2002) Humans integrate visual and haptic information in a statistically optimal fashion. Nature 415:429–433. 10.1038/415429a 11807554

[B16] Fengler A, Govindarajan LN, Chen T, Frank MJ (2021) Likelihood approximation networks (LANs) for fast inference of simulation models in cognitive neuroscience. Elife 10:e65074. 10.7554/eLife.65074 33821788 PMC8102064

[B17] Fortin C (2003) Attentional time-sharing in interval timing. In: Functional and neural mechanisms of interval timing (Meck WH, ed), pp 235–260. Boca Raton, FL: CRC Press.

[B18] Hartanto TA, Krafft CE, Iosif AM, Schweitzer JB (2016) A trial-by-trial analysis reveals more intense physical activity is associated with better cognitive control performance in attention-deficit/hyperactivity disorder. Child Neuropsychol 22:618–626. 10.1080/09297049.2015.1044511 26059476 PMC4675699

[B19] Hartcher-OBrien J, Luca MD, Ernst MO (2014) The duration of uncertain times: audiovisual information about intervals is integrated in a statistically optimal fashion (Balasubramaniam R, ed). PLoS One 9:e89339. 10.1371/journal.pone.0089339 24594578 PMC3942383

[B20] Iwasaki M, Noguchi Y, Kakigi R (2018) Neural correlates of time distortion in a preaction period. Hum Brain Mapp 40:804–817. 10.1002/hbm.24413 30276935 PMC6865754

[B21] Lemoine L, Lunven M, Bapst B, Cleret de LL, de Gardelle V, Bachoud-Lévi AC (2021) The specific role of the striatum in interval timing: the Huntington’s disease model. Neuroimage Clin 32:102865. 10.1016/j.nicl.2021.102865 34749287 PMC8569718

[B22] McAuley JH, Marsden CD (2000) Physiological and pathological tremors and rhythmic central motor control. Brain 123:1545–1567. 10.1093/brain/123.8.1545 10908186

[B23] Merchant H, Yarrow K (2016) How the motor system both encodes and influences our sense of time. Curr Opin Behav Sci 8:22–27. 10.1016/j.cobeha.2016.01.006

[B24] Mioni G, Grassi M, Tarantino V, Stablum F, Grondin S, Bisiacchi PS (2016) The impact of a concurrent motor task on auditory and visual temporal discrimination tasks. Atten Percept Psychophys 78:742–748. 10.3758/s13414-016-1082-y 26965441

[B25] Oldfield RC (1971) The assessment and analysis of handedness: the Edinburgh inventory. Neuropsychologia 9:97–113. 10.1016/0028-3932(71)90067-4 5146491

[B26] Palmer J, Huk AC, Shadlen MN (2005) The effect of stimulus strength on the speed and accuracy of a perceptual decision. J Vis 5:376–404. 10.1167/5.5.1 16097871

[B27] Paton JJ, Buonomano DV (2018) The neural basis of timing: distributed mechanisms for diverse functions. Neuron 98:687–705. 10.1016/j.neuron.2018.03.045 29772201 PMC5962026

[B28] Pedrosa DJ, Nelles C, Maier F, Eggers C, Burghaus L, Fink GR, Wittmann M, Timmermann L (2016) Variance of essential tremor patients’ time reproduction deficits. Mov Disord 31:1428–1429. 10.1002/mds.26736 27501132

[B29] Petzschner FH, Glasauer S, Stephan KE (2015) A Bayesian perspective on magnitude estimation. Trends Cogn Sci 19:285–293. 10.1016/j.tics.2015.03.002 25843543

[B30] Pineda JA (2005) The functional significance of mu rhythms: translating “seeing” and “hearing” into “doing”. Brain Res Rev 50:57–68. 10.1016/j.brainresrev.2005.04.005 15925412

[B31] Ratcliff R (1978) A theory of memory retrieval. Psychol Rev 85:59–108. 10.1037/0033-295x.85.2.59

[B32] Ratcliff R (1993) Methods for dealing with reaction time outliers. Psychol Bull 114:510–532. 10.1037/0033-2909.114.3.510 8272468

[B33] Robbe D (2023) Lost in time: relocating the perception of duration outside the brain. Neurosci Biobehav Rev 153:105312. 10.1016/j.neubiorev.2023.105312 37467906

[B34] Rohenkohl G, Cravo AM, Wyart V, Nobre AC (2012) Temporal expectation improves the quality of sensory information. J Neurosci 32:8424–8428. 10.1523/JNEUROSCI.0804-12.2012 22699922 PMC4235252

[B35] Ross JM, Comstock DC, Iversen JR, Makeig S, Balasubramaniam R (2022) Cortical mu rhythms during action and passive music listening. J Neurophysiol 127:213–224. 10.1152/jn.00346.2021 34936516 PMC8794057

[B36] Schütt HH, Harmeling S, Macke JH, Wichmann FA (2016) Painfree and accurate Bayesian estimation of psychometric functions for (potentially) overdispersed data. Vision Res 122:105–123. 10.1016/j.visres.2016.02.002 27013261

[B37] Shi Z, Church RM, Meck WH (2013) Bayesian optimization of time perception. Trends Cogn Sci 17:556–564. 10.1016/j.tics.2013.09.009 24139486

[B38] Singh A, Cole RC, Espinoza AI, Evans A, Cao S, Cavanagh JF, Narayanan NS (2021) Timing variability and midfrontal 4 Hz rhythms correlate with cognition in Parkinson’s disease. NPJ Parkinsons Dis 7:14. 10.1038/s41531-021-00158-x 33589640 PMC7884691

[B39] Smith A, Taylor E, Rogers JW, Newman S, Rubia K (2002) Evidence for a pure time perception deficit in children with ADHD. J Child Psychol Psychiatry 43:529–542. 10.1111/1469-7610.00043 12030598

[B40] Tsao A, Yousefzadeh SA, Meck WH, Moser MB, Moser EI (2022) The neural bases for timing of durations. Nat Rev Neurosci 23:646–665. 10.1038/s41583-022-00623-3 36097049

[B41] van Wassenhove V, Buonomano DV, Shimojo S, Shams L (2008) Distortions of subjective time perception within and across senses. PLoS One 3:e1437. 10.1371/journal.pone.0001437 18197248 PMC2174530

[B42] Vicario CM, Martino D, Spata F, Defazio G, Giacchè R, Martino V, Rappo G, Pepi AM, Silvestri PR, Cardona F (2010) Time processing in children with Tourette’s syndrome. Brain Cogn 73:28–34. 20189281 10.1016/j.bandc.2010.01.008

[B43] Voss A, Lerche V, Mertens U, Voss J (2019) Sequential sampling models with variable boundaries and non-normal noise: a comparison of six models. Psychon Bull Rev 26:813–832. 10.3758/s13423-018-1560-4 30652240 PMC6557879

[B44] Voss A, Rothermund K, Voss J (2004) Interpreting the parameters of the diffusion model: an empirical validation. Mem Cognit 32:1206–1220. 10.3758/bf03196893 15813501

[B45] Wiecki TV, Sofer I, Frank MJ (2013) HDDM: hierarchical Bayesian estimation of the drift-diffusion model in Python. Front Neuroinform 7:14. 10.3389/fninf.2013.00014 23935581 PMC3731670

[B46] Wiener M, Thompson JC, Coslett HB (2014) Continuous carryover of temporal context dissociates response bias from perceptual influence for duration. PLoS One 9:e100803. 10.1371/journal.pone.0100803 24963624 PMC4071004

[B47] Wiener M, Zhou W, Bader F, Joiner WM (2019) Movement improves the quality of temporal perception and decision-making. eNeuro 6:ENEURO.0042-19.2019. 10.1523/ENEURO.0042-19.2019 31395616 PMC6709222

