# Supplementary figures and images for: Mechanically Induced Motor Tremors Disrupt the Perception of Time
Source: eNeuro. 2024 Sep 13;11(9):ENEURO.0013-24.2024. doi: 10.1523/ENEURO.0013-24.2024 (PMC11412164; doi:10.1523/ENEURO.0013-24.2024)

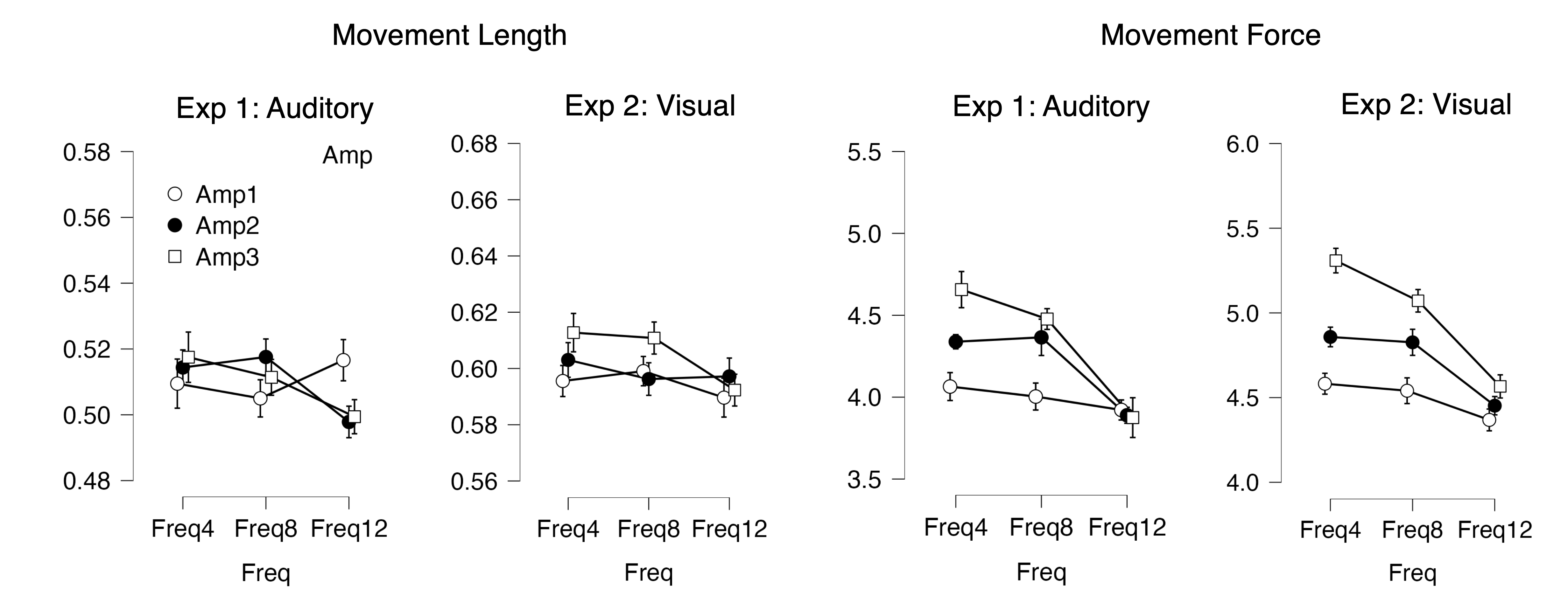

Supplement: Figure 2-1 — Movement length and force effects across both experiments. Download Figure 2-1, TIF file. [file eneuro-11-ENEURO.0013-24.2024-s002.tif]

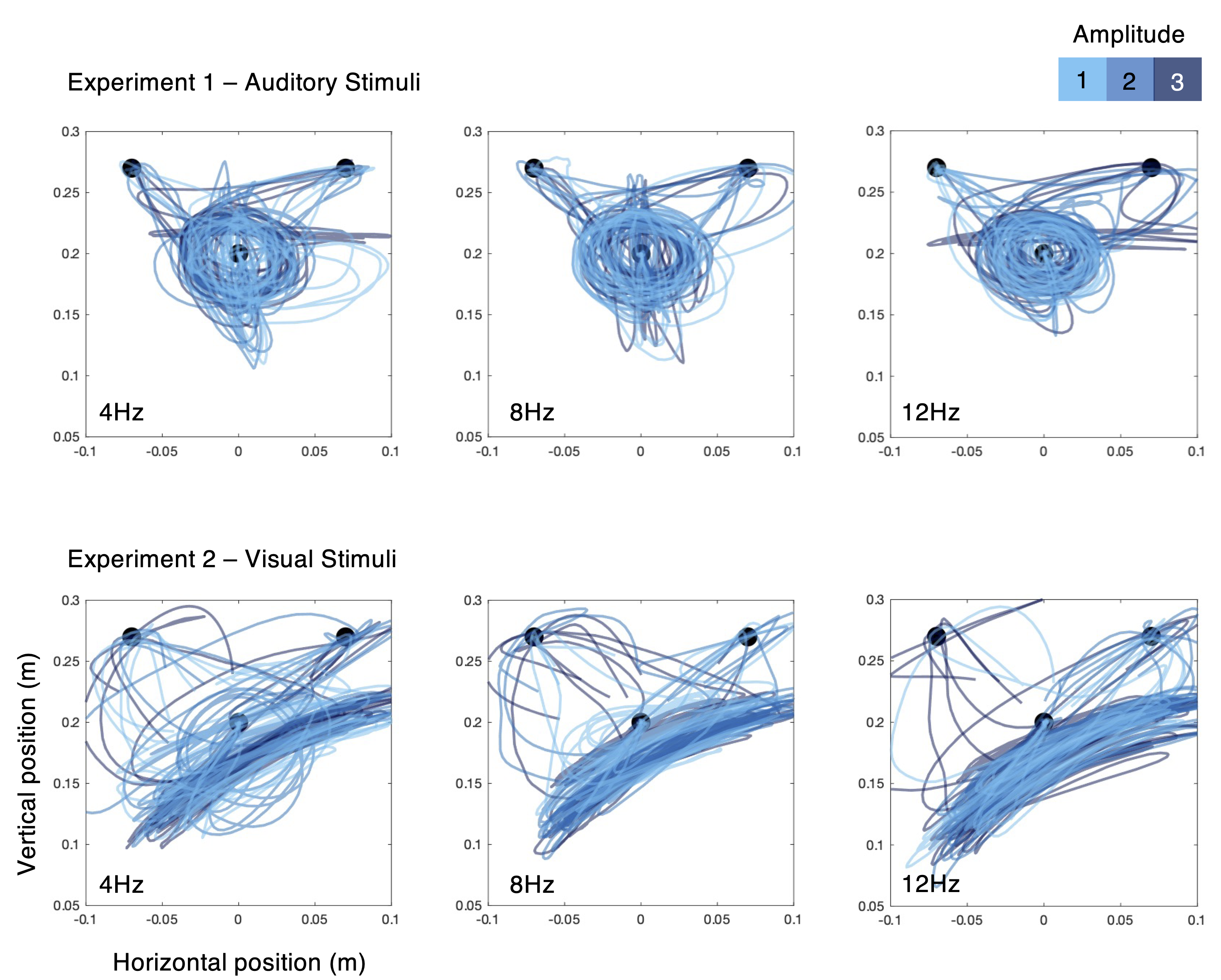

Supplement: Figure 2-2 — Example trajectories for two sample subjects from Experiments 1 (top) and 2 (bottom). Each panel displays all of the trajectories for the middle duration trials (2000ms). Center black point represents the starting zone whereas the upper two locations are the target response zones. Like previous reports, subjects adopted idiosyncratic yet consistent movement strategies during the task. Download Figure 2-2, TIF file. [file eneuro-11-ENEURO.0013-24.2024-s003.tif]
